# Diabetes, Cognitive Function and Mortality Risk Among Older Hispanics

**DOI:** 10.3390/brainsci15090988

**Published:** 2025-09-14

**Authors:** Jagdish Khubchandani, Elizabeth England-Kennedy, Srikanta Banerjee, Karen Kopera-Frye, Kavita Batra

**Affiliations:** 1 College of Health, Education, and Social Transformation, New Mexico State University, MSC 3AC, P.O. Box 30001, Las Cruces, NM 88003, USA; eengken@nmsu.edu (E.E.-K.); kfrye@nmsu.edu (K.K.-F.); 2 College of Health Sciences, Walden University, Minneapolis, MN 55401, USA; srikanta.banerjee2@mail.waldenu.edu; 3 Department of Medical Education, University of Nevada Las Vegas, Las Vegas, NV 89102, USA; kavita.batra@unlv.edu

**Keywords:** diabetes, cognition, mortality, Hispanic, dementia, brain health

## Abstract

**Background:** Diabetes is a leading cause of death globally and is strongly associated with aging-related conditions, such as cognitive decline. Both diabetes and cognitive impairment share overlapping biological mechanisms, including vascular damage and insulin resistance. While each condition independently affects health outcomes, the impact of their coexistence on mortality risk among older Hispanic adults remains understudied. This study aimed to examine the impact of the combination of diabetes and low cognitive performance on mortality risk among this marginalized population. **Methods:** Data were drawn from 636 Hispanic adults aged 60 years and older who participated in NHANES 1999–2002, with mortality follow-up through 2019. Cox proportional hazards models were used to estimate hazard ratios (HRs) for all-cause mortality, adjusting for sociodemographic and health-related variables. **Results:** Among participants, 23.3% had diabetes, and 54.9% had low cognitive performance. The combination of diabetes and low cognitive performance was associated with a significantly elevated risk of all-cause mortality (HR = 2.36; 95% CI: 1.70–3.28). No statistically significant increase in mortality risk was observed for either condition alone (i.e. diabetes or cognitive impairment). **Conclusions:** Coexisting diabetes and cognitive impairment in older Hispanic adults were associated with more than a twofold increase in mortality risk. These findings underscore the need for culturally appropriate, interdisciplinary strategies to address the dual burden of diabetes and cognitive decline in aging minority populations.

## 1. Background

Diabetes is a leading cause of death globally, with more than 1.5 million deaths per year directly related to the disease. Additionally, millions of deaths are indirectly related to diabetes, primarily from kidney and cardiovascular disease (e.g., stroke, myocardial infarction, and chronic kidney disease) [1,2,3]. Recent estimates suggest that nearly a tenth of adults worldwide live with diabetes, and it is projected that the prevalence of diabetes will increase by 25% in 2030 and by 50% in 2045 [2,3,4]. In the United States (U.S), more than 38 million adults (equating to nearly 15% of the population) have diabetes, with groups such as Hispanics having disproportionately higher rates. Hispanics have higher rates of complications and mortality from diabetes compared to non-Hispanic Whites [4,5,6,7]. Diabetes risk also increases substantially with age, and the incidence rises sharply during the fourth decade of life, with a continually increasing burden among older age groups. For example, in the U.S., adults above the age of 60 years have the highest rates of diabetes, affecting more than a quarter of individuals in this age group [5,6,7]. Such age-related risk of diabetes has been attributed to insulin resistance caused by cellular changes (e.g., increased oxidative stress, systemic low-grade inflammation, and cellular senescence), body composition changes (e.g., increased abdominal fat and decreased lean muscle mass), and age-related physiological changes (e.g., reduced insulin sensitivity, lower pancreatic beta cell efficiency, and sarcopenia) [8,9,10].

Another age-related problem that frequently co-occurs with diabetes is cognitive decline, a broad term that refers to effects on a wide range of areas, including memory, learning, attention, and executive function. Deterioration in these domains leads to challenges in comprehending or recalling information among those with cognitive decline [11,12,13]. Diabetes can lead to cognitive decline through numerous physiological and pathogenetic mechanisms [10,11,12,13,14,15,16,17,18,19,20]. For example, chronically elevated blood sugar can damage blood cells and vessels throughout the body, including those in the brain. This results in a reduction in blood flow and oxygen to brain tissue. Such vascular dysfunction is especially detrimental for brain regions associated with memory and executive functions. Furthermore, insulin resistance related to diabetes directly impacts brain tissue, which possesses its own insulin-signaling pathway that is essential for memory formation and synaptic activity. Diabetes also fosters chronic inflammation and oxidative stress, both of which can harm brain cells and disrupt neuronal communication [14,15,16,17,18]. In addition, episodes of either excessively high or low blood sugar can temporarily impair cognitive abilities. Over time, damage caused by recurrent episodes can lead to enduring cognitive alterations, particularly impacting attention, processing speed, and memory. Chronic conditions and risk factors that are comorbid with diabetes (e.g., heart disease) can influence cognitive function as well [16,17,18,19], potentially compounding the effects of both. Social determinants of health (SDOH) serve as risk factors that are synergistic and accentuated in people with unfavorable SDOH. For example, in the U.S, older adults who are members of racial minority groups (e.g., Hispanics), have lower income and education, /or have inadequate access to healthcare, are at elevated risk of diabetes related complications, comorbid conditions, and cognitive decline [18,19,20]. Recent estimates suggest that older Hispanics are 1.3 to almost 2.0 times more likely than white older adults to have diabetes or cognitive decline; this equates to more than 10% older Hispanics having cognitive decline and more than 20% having diabetes [5,6,7,11,12,13,16,17,18]

A range of factors contribute to the increased risk of developing both diabetes and cognitive decline, including elevated body mass index (BMI), lack of physical activity, poor dietary habits, cardiovascular diseases, and specific genetic predispositions [16,17,18,19,20,21,22]. This overlap complicates the task of distinguishing between the directionality of the relationship, causal links, and shared underlying factors associated with diabetes and cognitive decline. For instance, cognitive decline can lead to impediments in the effective management of diabetes. Individuals experiencing cognitive impairments might struggle to follow medication schedules, monitor their blood glucose levels, access healthcare services, maintain physical activity, make appropriate dietary choices, and manage their diabetes effectively, potentially leading to a deterioration in diabetes control [21,22,23,24,25,26]. Still, the vast majority of the evidence points out to be one direction of causation (i.e., diabetes leading to cognitive decline). A meta-analysis of more than 100 prospective studies found that diabetes conferred a 1.25–1.91-fold excess risk for cognitive disorders (i.e., cognitive impairment and dementia) [24]. Another meta-analysis with more than 6000 participants found that the risk of progression from Mild Cognitive Impairment (MCI) to dementia among people with diabetes with a longer duration of diabetes was 1.53 times higher (95% CI 1.20–1.97) than those with a shorter duration, and that having diabetes-related complications increased the risk of progression of cognitive impairments [25]. Alternatively, diabetes control strategies (e.g., medication usage, continuous glucose monitoring, and lifestyle alterations) have been shown to reduce the risk of cognitive impairments [8,9,10,11,12,13,14,15,16,17,18,26]. Despite the well-established risk of cognitive impairment in those with diabetes, the long-term impact of cognitive impairment in those with diabetes is not well explored. Thus, through this investigation, we aimed to assess the mortality risk of those with diabetes based on cognitive performance. For this analysis, we selected a nationwide random sample of adults from a high-risk group (i.e., older Hispanics).

## 2. Methods

### 2.1. Study Participants and Procedures

The National Health and Nutrition Examination Survey (NHANES) data from 1999 to 2002 were analyzed for this study. NHANES data is collected by the National Center for Health Statistics (NCHS) as a part of an annual nationwide study to assess the health of individuals in the U.S using a survey, physical exam, and biomarker measurement; it employs a stratified, multistage, and cluster sampling methodology to acquire a representative sample of the noninstitutionalized civilian population in the United States [7,18,20,27]. The subjects are de-identified and assigned unique sequence numbers. NCHS suggests that to achieve enhanced statistical reliability, it is beneficial to merge two or more 2-year cycles of continuous NHANES data. Additionally, it is recommended to utilize the four-year weights, which were generated from the NHANES data collected between 1999 and 2002. The four-year sample weight is provided with the public use data files for individuals who were part of the study from 1999 to 2002, and this weight was employed for the purpose of weighing the variables. The total number of participants for NHANES 1999–2002 was 21,004. We restricted our sample to Hispanic individuals 60 years or older, given that they are disproportionately more likely to be affected by diabetes and due to a lack of research on this population. Furthermore, we included these participants only if they had complete data on cognitive function and diabetes. Vital status was ascertained by linking the NHANES 1999–2002 data with public-use mortality files from the National Death Index (from the date of survey participation through 31 December 2019). Data is available for public use through the CDC website, and details about this data have been extensively published [27,28,29,30,31,32,33]. The procedures and protocols for NHANES were approved by the NCHS before data collection.

### 2.2. Measures

To evaluate cognitive function, the Digit Symbol Substitution Test (DSST) was conducted among older adults as part of the NHANES survey from 1999 to 2002. This test is included in the Wechsler Adult Intelligence Test. The DSST measures response speed, sustained attention, visual-spatial abilities, and associative learning and memory. We categorized the results into two groups: the bottom 25th percentile, which indicates low cognitive performance, and the top 75th percentile. Comprehensive information regarding the administration of these tests and the performance metrics within NHANES is available in other sources [31,32,33,34]. All adult participants were asked, “Other than during pregnancy, have you ever been informed by a doctor or other health professional that you have diabetes or sugar diabetes?” For this study, those who responded with “borderline” or “yes” were classified as having diabetes [28,29,32].

The NHANES survey also includes numerous other health and sociodemographic data variables that were included in this analysis [32,33,34,35,36,37]. Cardiovascular disease history was determined by the self-reported diagnosis of coronary heart disease, angina, stroke, congestive heart failure, or myocardial infarction. For chronic kidney disease (CKD), the glomerular filtration rate was derived from the Cockcroft-Gault equation using measured creatinine levels to classify individuals into CKD levels. Data on body mass index (BMI) were derived from measured height and weight and categorized into four groups: BMI < 25 kg/m^2^ = normal weight; BMI = 25–29 kg/m^2^ = overweight; BMI = 30–39.9 kg/m^2^ = obese; and BMI > 40 kg/m^2^ = severely obese. For the multivariate models, this was dichotomized with participants being categorized as obese or not obese (cutoff for BMI ≥ 30 kg/m^2^).

A two-variable indicator of current smoking status was created with “nonsmoker” (coded 0) and “smoker” (coded 1). For education level, data on participants were categorized into three groups: “less than high school” versus “high school graduate,” versus “some college or above.” Age was treated as a continuous variable with mean and standard error calculations. Data was also collected in NHANES for the marital status of the participants, with response options falling into one of the six categories: married, living with a partner, never married, separated, divorced, or widowed. These were finally dichotomized to “married or living with a partner” versus “other”. Income information was computed by poverty-income-ratio (PIR), which is also an indicator of income relative to the economic needs of a household (i.e., the ratio of income to the family’s appropriate poverty threshold as determined by family size and composition). PIR levels were defined as low income (PIR < 1), middle income (1 ≤ PIR < 4), and high income (PIR ≥ 4), and dichotomized for analysis with a cutoff point of 1 [34,35,36,37].

### 2.3. Statistical Analysis

Multiple techniques were used to ensure data analysis as per the recommendation of the NCHS [27,28,29,30,31,32]. First, descriptive statistics were computed to describe the study population (percentage and 95% confidence intervals). Second, group differences were assessed for those with and without diabetes and based on cognitive function by utilizing Chi-Square tests. Third, hazard ratios were computed by constructing multiple Cox regression models to examine the risk of mortality based on diabetes or cognitive functioning. The dependent variable for this study was all-cause mortality, while diabetes and cognitive function served as independent variables. In accordance with the analytical guidelines from the NCHS, the study variables were weighted to approximate distributions in the US by using the provided sample weights (to account for oversampling of certain groups, unequal probabilities of selection, and non-response). The Taylor series linearization variance estimation method was used for all analyses [28,29,30,31,32,33,34,35]. Statistical analyses were conducted using the SAS System for Windows (release 9.3; SAS Institute Inc., Cary, NC, USA) and SUDAAN (release 9.0; Research Triangle Park, NC, USA). Statistical significance for tests was considered at a *p*-level of <0.05.

## 3. Results

A total of 636 Hispanic adults aged ≥60 years were included in the final analysis, where more than a fifth (23.28%) had diabetes and slightly more than half (54.88%) had low cognitive performance. The average age of the study participants was 69.9 years, and more than a third were males (38.9%). Table 1 provides data on the distribution of demographic characteristics of the study participants stratified by cognitive performance and diabetes using bivariate analysis. Individuals with lower cognitive performance were statistically significantly more likely to be older, male, have lower education and income, be separated or never married, and have a history of CVD or CKD, but were less likely to be obese or smokers. In comparison, those with diabetes were statistically significantly more likely to have lower education, be unmarried/ living without a partner, have higher BMI, and have a history of CVD or CKD (Table 1).

Irrespective of cognitive performance, compared to those without diabetes, the adjusted hazard ratio (HR) for all-cause mortality among those with diabetes was significantly elevated [HR = 1.38, 95% CI = 1.16 − 1.65, *p* < 0.05; Table 2, column 2]. This relationship between diabetes and mortality risk was moderated by age, gender, and history of CVD. When stratified by groups based on diabetes or cognitive performance, the adjusted HR for mortality risk was highest among individuals with both diabetes and low cognitive performance [HR= 2.36 (95%CI = 1.70–3.28), *p* < 0.01]. However, among individuals with diabetes only [HR = 0.81, 95% CI = 0.55–1.07] or low cognitive performance only [HR = 0.72, 95% CI = 0.35–1.08], the risk of mortality was not statistically significantly elevated. Across all the comparison groups, age and history of CVD were consistently significant moderators of the relationship between diabetes or cognitive performance and the risk of mortality.

## 4. Discussion

In this nationwide assessment of older Hispanics, diabetes or cognitive impairment alone did not increase the risk of mortality after adjustment for numerous demographic and health-related variables. However, among individuals with both diabetes and cognitive impairments, the risk of any cause of death increased more than twofold. This is in contrast to another large study on this topic, which found that diabetes or cognitive impairments individually can also increase the risk of mortality (along with the higher risk of mortality among those with both diabetes and cognitive impairments). However, this study was based on data from 4499 older adults in one province of China, did not use DSST, and the analysis was not adjusted for critical factors such as income or CKD history (a major health risk among those with diabetes) [38]. Another study of 559 American adults ≥ 70 years old found that individuals with diabetes and low levels of cognition were approximately 20% more likely to die and 13% more likely to become disabled than those with higher levels of cognitive functioning over 2 years. Again, this study did not adjust for BMI, smoking status, income, or CKD history [39]. Similarly, a study by Martinez and colleagues using the U.S. Health and Retirement Study data on Hispanics found that cognitive impairment is linked with increased mortality among those with or without diabetes. However, this study included adults above the age of 50 years and did not account for variables such as BMI and CKD [12]. Despite these differences, our findings are novel and have major implications for research, clinical practice, and prevention, given the high burden of diabetes among Hispanic adults and the rising prevalence of cognitive decline among aging populations.

While certain pathophysiological pathways can explain the relationship between diabetes, cognitive function, and mortality, additional research is needed to dissect these relationships. For example, as age was an influential variable in the relationship between diabetes, cognitive decline, and mortality, it could be possible that diabetes and cognitive decline together could accelerate aging, leading to premature mortality. A study of 13,687 older adults found that diabetes reduced total life expectancy by 5–7 years and cognitively healthy life expectancy by 4–6 years, and those with diabetes lived one year less in a cognitively impaired state than those without diabetes [40]. Furthermore, as we found that CVD history was an influential variable, both diabetes and cognitive impairment are associated with CVD or its risk factors; a conglomeration of these factors could lead to premature mortality. A recent study of 2564 American adults aged ≥60 years found that the individual impact of diabetes or CVD on cognitive function was not significant, but cognitive impairment risk was increased when both diseases coexisted [41]. Additional explanations for our findings lie in the fact that people with cognitive impairments may have frequent episodes of hyperglycemia or hypoglycemia due to an inability to monitor or manage blood sugar, have adequate and timely access to care, adhere to medical recommendations, exercise, or eat healthily, all of which could increase the risk of mortality. Also, individuals with cognitive impairments may have a higher risk of complications of diabetes (e.g., falls or infections), institutionalization, profound disability, psychiatric illnesses, or problems managing multiple comorbidities that occur with aging, further increasing risk of mortality among those with diabetes [24,25,39,40,41,42,43,44]. Systemic inflammation, vascular dysfunction, impaired neurogenesis, altered neurotransmitter release, endothelial damage, and disruption of cellular metabolism have been cited as potential underlying biological pathways for the higher risk of mortality among those with diabetes or cognitive impairments [9,14,15,24,25]. Despite these explanations, additional research is needed with larger and more diverse population samples to understand the nature (i.e., cause of death) and extent of mortality risk among those with diabetes and cognitive impairments.

Experts suggest that while the clinical management of diabetes or cognitive impairments among older adults is often difficult, the management of both these problems occurring together is extremely challenging. Further complicating the management of these dual disorders is the type of population we examined: older ethnic minorities who face numerous other unfavorable SDOH and deprivation [8,10,15,17,19]. Still, there are numerous practical and actionable steps to be taken by clinicians and caregivers of those with diabetes and cognitive impairments. First, given the unique nature of this population (i.e., older, ethnic minority, and cognitive impairments), much focus needs to be on frequent and culturally/linguistically appropriate communication through multiple modes (e.g., caregivers, text messages, and the use of heritage-language-speaking community health educators who are certified in specific health conditions) [17,19,20,44,45,46]. Second, the approach to management should always include an interdisciplinary team of healthcare professionals, including endocrinologists, neurologists, geriatricians, dietitians, physical therapists, nurses, and social workers, to provide comprehensive and coordinated care. Clinicians and the care team need to be aware of the power of culturally tailored, evidence-based treatment regimens and interventions when motivating diverse populations for behavioral change in managing chronic conditions [22,42,43,45,47]. Third, clinicians should plan a comprehensive and individualized management plan for optimizing glycemic control (e.g., strict monitoring and control of blood sugar with technology usage or caregiver support, frequent medication review and dose adjustment with balancing for the risks of hyperglycemia and hypoglycemia, etc.). In line with this, there should be aggressive management of CVD risk factors (e.g., blood cholesterol or pressure) as CVDs are a major driver of diabetes, cognitive dysfunction, or premature mortality [8,10,41,45,46,47,48,49]. Fourth, there is a need to increase minority older adults’ adherence to preventative screenings as per guidelines from professional organizations. These would include screenings for cognitive function, mental health, diabetes complications, polypharmacy, vaccinations, eating difficulties, functional limitations, sleep hygiene, and other relevant health situations and behaviors [8,10,15,17,19,46,47,48,49]. Fifth, lifestyle interventions and alterations should be prescribed to patients or their caregivers. These would include increasing exercise, balance and strength training, nutritionally sound meals, consistent food consumption and access, mentally engaging activities, cognitive stimulation and training, and social engagement, among others [45,47,48,49,50,51]. Sixth, a person-centered and caregiver-supported approach should be instituted for those with both diabetes and cognitive impairments. These could include caregiver education (e.g., diabetes and dementia training), family support (e.g., respite groups to prevent caregiver burnout), and individualized care plans (e.g., plans that consider patient cognitive abilities, preferences, comorbidities, and overall goals of care) [50,51,52,53].

Beyond these strategies, cognitive decline and diabetes cannot be managed in isolation with a marginalized population like older Hispanics. Other contextual factors also need to be considered [52,53,54,55,56,57]. Addressing unfavorable social determinants of health (SDOH) is critical for ethnic minority older adults with diabetes and cognitive decline and may require a multi-level strategy that blends clinical care with community empowerment and policy change. At the community level, there is a need to address the social needs of this population through culturally informed outreach (e.g., to address gaps in care, provide transportation, and ensure food security with an emphasis on culturally preferred foods). At the clinical level, there should be greater screening for SDOH (particularly socioeconomic and access-related needs), and implementation of integrated care models to address these needs and manage patient care (e.g., appointments, medications, insurance coverage, and community resources and benefits). Policy-level interventions could address housing and food insecurity among these populations, expansion of healthcare access, reimbursement for community health workers and social care coordination, and greater fiscal coverage (e.g., for technological devices, home healthcare, counseling for nutrition, and transportation) [24,25,26,51,52,53,54,55,56,57].

Despite its novelty and strengths, this study and its findings are subject to potential limitations. First, except for mortality data, the majority of the information in this study was derived from self-reported data, which could limit the reliability of findings. For example, we could have used blood sugar or HbA1c data from NHANES as well, but this would have heavily reduced and limited the sample size due to lack of extensive biomarker information for older Hispanics (whereas self-reported diabetes among older Hispanics comprised a larger sample size). Also, the results are subject to all limitations of reliability and validity inherent to cross-sectional study designs. Importantly, the diagnosis of diabetes and cognitive function needs further validation in future studies to compute true estimates of these disorders and their relationship with mortality risk. Second, potentially pertinent information regarding diabetes (e.g., duration, severity, and treatments taken) and cognitive impairment (e.g., disability and duration) was not available in detail in the NHANES; such detailed information is needed for further investigation of the relationships examined in this study. Third, we examined all-cause mortality risk, but there could be differences in mortality risks from various causes, and future studies should elaborate on precise causes of death (e.g., mortality from CVD or respiratory failure) among those with diabetes and cognitive impairments. Fourth, while we adjusted our analysis for numerous sociodemographic and health-related variables, residual confounding cannot be ruled out (e.g., due to factors such as physical activity, diet, genetic predisposition to dementia, or other factors like depression, which could lead to cognitive dysfunction, etc.). Fifth, even though NHANES data are widely used and considered representative of various groups of the U.S. population, caution should be exercised when generalizing our findings to other populations. Future longitudinal studies with larger samples and variations of Hispanic older adult populations should account for these limitations, include more specific biomarker data, and estimate the risk of mortality using longer follow-up periods among those with diabetes, cognitive decline, or both of these.

## 5. Conclusions

Hispanic older adults have higher rates of morbidity and mortality than their non-Hispanic White peers and also experience higher rates of health risk factors and co-morbidities, such as diabetes and cardiovascular disease. Cognitive decline that is comorbid with diabetes can increase the risk of further chronic disease and other morbidity, as well as the likelihood of premature death. In this study of older Hispanics, we found that compared to those without diabetes, the adjusted hazard ratio (HR) for all-cause mortality among those with diabetes was significantly elevated; it was moderated by age, gender, and history of CVD. Also, the risk of mortality was highest among individuals with both diabetes and low cognitive performance. For older Hispanics with diabetes, there is a need to employ interprofessional care teams that utilize culturally and linguistically appropriate communication through multiple modes and caregiver-supported approaches, comprehensive and individualized health management plans, adherence to professionally recommended and regular evaluations, the inclusion of SDOH in screening procedures, and policies that support increased access to care and preventive services to reduce mortality due to factors such as cognitive impairment. Future research on pathophysiological pathways connecting diabetes, cognitive function, and mortality is needed, particularly with larger and more diverse population samples. Longitudinal studies with detailed data on diabetes duration, severity, treatment, and cognitive trajectories are particularly needed. Research on the impacts of additional factors such as age and level of education, impacts of policy, and of integrating cultural values and practices into health promotion programs, and on the combined and severable impacts of cognitive decline and diabetes is also needed.

## Figures and Tables

**Table 1:** Characteristics of Hispanic Study Participants Stratified by Cognitive Functioning and Diabetes.

Characteristics	Total Population (*n* = 636)	Low Cog. Perf. (*n* = 349)	Normal Cog. Perf. (*n* = 287)	Diabetes (Yes) (*n* = 148)	Diabetes (No) (*n* = 473)
**Age** (Mean ± S.E.)	69.9 (0.31)	70.8 (0.47) *	67.2 (0.61)	69.4 (0.64)	70.0 (0.36)
**Gender, Male** (%)	38.9 (35.9–41.9)	43.2 (38.0–48.3)	34.7 (27.9–41.5)	34.9 (29.2–40.6)	41.2 (39.0–43.3)
**Education Level (%)**					
Some High School	57.8 (51.0–63.8)	81.7 (78.3–85.1) **	35.3 (23.1–47.5)	69.5 (62.9–76.1) *	61.3 (55.5–67.1)
High School Grad	15.4 (11.7–19.1)	11.2 (9.1–13.3)	19.4 (12.2–26.5)	7.9 (5.4–11.5)	16.0 (11.7–20.2)
Some College or Above	26.8 (19.3–34.3)	7.1 (5.4–9.4)	45.3 (29.8–60.8)	22.6 (16.9–28.3)	22.7 (15.2–30.3)
**Marital Status**					
Married	53.3 (50.0–56.7)	53.3 (48.2–58.4) *	53.4 (49.4–57.3)	49.2 (38.6–59.7) *	53.8 (49.9–57.8)
Widowed	22.9 (19.7–26.0)	19.8 (15.9–23.7)	25.9 (21.1–30.7)	26.1 (20.3–32.0)	24.8 (22.0–27.7)
Divorced	12.5 (9.8–15.2)	12.4 (8.3–16.4)	12.6 (7.8–17.4)	14.9 (9.9–19.9)	9.9 (7.1–13.6)
Separated	4.2 (2.4–7.3)	6.1 (3.0–11.9)	2.3 (1.2–4.5)	3.6 (2.2–5.7)	3.6 (1.9–7.0)
Never Married	6.6 (5.8–7.5)	8.3 (6.5–10.6)	4.8 (3.7–6.3)	5.9 (4.5–7.7)	7.3 (6.1–8.8)
Living with Partner	0.5 (0.2–1.2)	0.1 (0.1–0.8)	1.0 (0.4–2.4)	0.3 (0.1–2.4)	0.5 (0.2–1.1)
**Family Poverty Income Ratio** * (%) (PIR < 1)	39.8 (34.1–45.6)	48.0 (40.8–55.1) **	31.8 (25.3–38.2)	37.0 (29.9–44.1)	41.8 (35.6–47.9)
**Diabetes**	17.3 (12.7–21.9)	17.1 (11.8–22.4)	17.5 (12.1–22.9)	N/A	N/A
**Smoking**	16.1 (13.4–18.9)	13.0 (8.7–17.4) *	19.2 (14.5–23.9)	15.8 (11.1–20.6)	16.0 (13.0–19.0)
**Cardiovascular Disease** */**	16.1 (11.9–20.3)	21.1 (17.0–25.2) **	11.1 (5.0–17.3)	28.9 (24.3–33.5) **	14.1 (11.7–16.4)
**CKD**	19.2 (15.6–22.8)	25.6 (20.7–30.5) **	12.8 (6.5–19.1)	23.5 (18.3–28.8) *	21.4 (17.7–25.0)
**Obesity Status** (%)/*					
Normal weight	23.6 (18.4–28.7)	26.3 (17.9–34.7) **	21.1 (16.4–25.8)	20.7 (15.0–26.4) **	24.2 (19.2–29.2)
Overweight	47.2 (42.1–52.2)	45.1 (40.4–49.7)	49.1 (42.9–55.2)	48.2 (41.9–54.5)	47.6 (41.0–54.2)
Obese	27.2 (24.0–30.4)	26.2 (20.6–31.8)	28.1 (22.9–33.3)	26.1 (15.4–36.8)	27.0 (23.1–31.0)
Severely Obese	2.1 (1.4–3.1)	2.4 (1.7–3.5)	1.7 (0.8–3.5)	5.0 (3.4–7.4)	1.1 (0.59–2.1)
**All deaths** (N) **/*	348 (57.2%)	212 (60.4%)	136 (54.0%)	90 (62.1%)	252 (56.1%)

Note. * *p* < 0.05 ** *p* < 0.01. Numbers with 95 CI indicate 95% confidence intervals for proportions.

**Table 2:** Risk of Mortality Among Older Hispanics Based on Cognitive Functioning and Diabetes History.

	Total Population HR (95% CI) (Diabetes vs. No Diabetes)	Low Cog+ Diabetes- HR (95% CI)	Low Cog- Diabetes+ HR (95% CI)	Low Cognition+ Diabetes+ HR (95% CI)
**Diabetes & Cognitive Performance**	1.38 (1.16–1.65) *	0.72 (0.35–1.08)	0.81 (0.55–1.07)	2.36 (1.70–3.28) **
**Smoking Status** (ref: no)	0.90 (0.66–1.22)	0.73 (0.43–1.23)	0.46 (0.21–1.00)	1.35 (0.92–1.98)
**CVD** (ref: no)	1.56 (1.05–2.31) *	1.93 (1.16–3.21) *	2.59 (1.48–4.52) **	1.36 (1.08–1.96) *
**CKD** (ref: no)	0.95 (0.65–1.40)	1.07 (0.85–1.36)	0.83 (0.45–1.53)	1.45 (0.49–2.22)
**Obesity** (ref: no)	1.71 (0.94–3.12)	1.86 (1.11–3.10) *	1.76 (0.81–3.81)	1.32 (0.95–1.86)
**Age**	1.11 (1.06–1.17) *	1.13 (1.05–1.23) *	1.11 (1.10–1.22) *	1.12 (1.08–1.15) *
**Gender** (Ref: Female)	1.45 (1.04–2.02)*	1.79 (1.27–2.51)	1.45 (0.94–2.24)	2.00 (1.21–3.30)
**Education Level**				
Some college & beyond	Ref	Ref	Ref	Ref
Some High School	1.28 (0.75–2.17)	1.12 (0.52–2.42)	0.83 (0.37–1.86)	1.65 (0.87–3.12)
High School Graduate	1.35 (0.68–2.70)	0.98 (0.35–2.74)	1.43 (0.60–3.38)	1.60 (0.74–3.42)
**Marital Status** (Ref: never married/separated/divorced/widowed)	1.27 (0.69–2.33)	1.17 (0.65–2.10)	2.44 (1.43–4.14) *	0.96 (0.33–2.83)
**Family Poverty-Income-Ratio** (Ref: PIR ≥ 1)	0.79 (0.57–1.09)	0.85 (0.61–1.19)	0.88 (0.50–1.56)	0.86 (0.49–1.51)

Note. * *p* < 0.05 ** *p* < 0.01. HR (95 CI) indicates hazard ratios with 95% confidence intervals for the outcome (i.e., mortality). Ref indicates the reference group among each variable for comparison with other groups.

## Data Availability

Data files are publicly available through the National Center for Health Statistics (NCHS).
